# CO_2_/N_2_-Responsive Nanoparticles for Enhanced Oil Recovery During CO_2_ Flooding

**DOI:** 10.3389/fchem.2020.00393

**Published:** 2020-05-21

**Authors:** Nanjun Lai, Qingru Zhu, Dongyu Qiao, Ke Chen, Dongdong Wang, Lei Tang, Gang Chen

**Affiliations:** ^1^School of Chemistry and Chemical Engineering of Southwest Petroleum University, Chengdu, China; ^2^State Key Laboratory of Oil and Gas Geology and Exploitation of Chengdu University of Technology, Chengdu, China; ^3^Oil and Gas Field Applied Chemistry Key Laboratory of Sichuan Province, Chengdu, China; ^4^Engineer Technology Research Institute, CNPC Xibu Drilling Engineering Company Limited, Urumqi, China; ^5^China National Offshore Oil Corporation (CNOOC) Energy Development Company Limited, Tianjin, China

**Keywords:** responsive nano-SiO_2_, plugging, mobility control, enhanced oil recovery, CO_2_ flooding

## Abstract

During CO_2_ flooding, serious gas channeling occurs in ultra-low permeability reservoirs due to the high mobility of CO_2_. The chief end of this work was to research the application of responsive nanoparticles for mobility control to enhance oil recovery. Responsive nanoparticles were developed based on the modification of nano-silica (SiO_2_) by 3-aminopropyltrimethoxysilane (KH540) via the Eschweiler-Clark reaction. The proof of concept for responsive nanoparticles was investigated by FT-IR, ^1^H-NMR, TEM, DLS, CO_2_/N_2_ response, wettability, plugging performance, and core flooding experiments. The results indicated that responsive nanoparticles exhibited a good response to control nanoparticle dispersity due to electrostatic interaction. Subsequently, responsive nanoparticles showed a better plugging capacity of 93.3% to control CO_2_ mobility, and more than 26% of the original oil was recovered. Moreover, the proposed responsive nanoparticles could revert oil-wet surfaces to water-wet, depending on surface adsorption to remove the oil from the surface of the rocks. The results of this work indicated that responsive nanoparticles might have potential applications for improved oil recovery in ultra-low permeability reservoirs.

## Introduction

With the continuous exploitation of conventional reservoirs, production capacity is gradually becoming exhausted. More and more researchers have turned their attention to the development of ultra-low permeability reservoirs. Nevertheless, due to their ultra-low permeability, such reservoirs have a tendency to present some extraordinary features, such as a small pore throat and strong heterogeneity (Wang et al., 2015, 2017). In view of this, carbon dioxide (CO_2_) flooding is a promising technology for enhanced oil recovery (EOR) (Zhang et al., 2018; Jia et al., 2019; Zhou et al., 2019). CO_2_ can dissolve into oil to reduce the oil–water interfacial tension and oil viscosity, which improves the mobility ratio during CO_2_ flooding. Moreover, CO_2_ resources are abundant, and CO_2_ is non-toxic. However, while CO_2_ flooding can be a highly effective technology for EOR in ultra-low permeability reservoirs, it comes with certain limitations, including gas channeling caused by the high mobility of CO_2_. The heterogeneous characteristic of the reservoir makes this condition even worse (Abedini and Torabi, 2014; Gong and Gu, 2015; Yu et al., 2015), leading to low sweep efficiency.

The conventional methods utilized to mitigate CO_2_ gas channeling are gel injection, polymer injection, and foam injection. Li et al. (2016) proposed the modification of polyacrylamide-methenamine-resorcinol gel agents to block CO_2_ channeling. Sun et al. (2016) reported that aqueous foams stabilized by partially hydrophobic SiO_2_ showed better CO_2_ mobility control performance. Li et al. (2017) investigated the efficacy of foam prepared from an organic amine, octadecyl dipropylenetriamine, for blocking gas breakthrough channeling and mobility control. Barrabino et al. (2018) showed that a graphene oxide foam system achieved immediate gel formation, which improved foam stability and caused particles to block pores for mobility control. Lu et al. (2019) showed that the interaction of hydrophobically modified polyacrylamide (HMPAM) with fatty alcohol improved the efficiency of mobility control. However, according to the bridge principle (Lai et al., 2016), the molecular diameter of gel or polymers (4–16 μm) is incompatible with the ultra-low reservoir pore throat radius (0.5–2 μm) (Dongqi et al., 2019). For foam injection, the stability and strength of foam systems with high apparent viscosity are usually not sufficient to plug the gas channeling. Hence, it is urgent to find and prepare a new material that satisfies the injection requirements of ultra-low permeability reservoirs and improves CO_2_ mobility control.

In recent years, nano oil displacement technology has been developed continuously. Functional nanoparticles have been applied in aspects such as wettability, surfactivity, pressure decrease, and augmented injection because of the unique properties of nanoscale particles. Sharma et al. (2014), Dai et al. (2015), Zhang H. et al. (2016), Emrani and Nasr-El-Din (2017), and Li et al. (2019). In addition, the modification of the surfaces of SiO_2_ nanoparticles with pH-responsive, light-responsive, and CO_2_/N_2_-responsive groups has also been reported increasingly (Jiang J. et al., 2016; Jiang W. et al., 2016; Yan et al., 2018). The structure of the tertiary amine group as a responsive group causes it to have better effects on CO_2_/N_2_ response compared with the amidine group. For example, Zhang Y. et al. (2016) reported on a CO_2_-responsive Pickering emulsion prepared from nano-silica particles modified by extensive hydrophobic tertiary amine. Zhang et al. (2019) proposed a CO_2_-responsive wormlike micelle (WLM) system that generated bulk gel to block gas channeling for EOR. Liu et al. (2017) reported the synthesis of CO_2_-switchable silica nanohybrids with tertiary amine via Michael addition of methyl acrylate and amidation reaction for enhancing CO_2_ flooding.

All of the above indicates that using tertiary amine to control system performance is a feasible method for EOR. The idea of grafting tertiary amine-functionalized short chains onto the surface of nano-SiO_2_ has been proposed. In order to achieve this, the Eschweiler-Clark reaction (Zhu and Sun, 2018) is a methylation method that can be utilized to synthesize tertiary amine from primary amines, and the conditions of the reaction are mild and simple. A well-known method of modifying the surface of nanoparticles to primary amine is the silane coupling agent method (Liu et al., 2015; Lai et al., 2017, 2019). Consequently, we endeavored to introduce a silane coupling agent to synthesize a modified nano-SiO_2_ and then to prepare responsive nano-SiO_2_ via the Eschweiler-Clark reaction. Subsequently, the CO_2_/N_2_ response and dispersibility of the responsive nanoparticles were investigated. A series of experiments were implemented to explore their feasibility for EOR in ultra-low permeable reservoirs, including plugging performance, core flooding experiments, and wettability measurements. Developing this application would certainly open up responsive nano-SiO_2_ as an important new frontier.

## Experiment

### Reagents and Materials

Sodium hydroxide (NaOH), polyethylene glycol 400 (PEG-400), methylbenzene (C_7_H_8_), 3–aminopropyltrimethoxysilane (KH−540), ethanol (C_2_H_5_OH), formic acid (HCOOH), formaldehyde (HCHO), N, N–dimethylformamide (DMF), and hydrochloric acid (HCl) were obtained from Chengdu Kelong Chemical Reagent Co., Ltd (Sichuan, China). All chemical reagents were analytical grade. Nano-SiO_2_ particles (10–20 nm) with a purity of >99.8% were purchased by Aladdin Chemistry Co., Ltd. (Shanghai, China). CO_2_ (g) and N_2_ (g) were purchased from Chengdu Jingli Gas Co., Ltd. (Sichuan, China). Cores with permeabilities from 1 × 10^−3^ μm^2^ to 10 × 10^−3^ μm^2^ were purchased from the Center for Well Completion and Logging Lab (Sichuan, China). A mixture of kerosene and dehydrated crude with a density of 0.7783 g/ cm^3^ formed the crude oil. Water was re-distilled and deionized through an ion-exchange column.

### Synthesis of Responsive Nano-SiO_2_ and Preparation of Nanofluid

**Figure 1:**
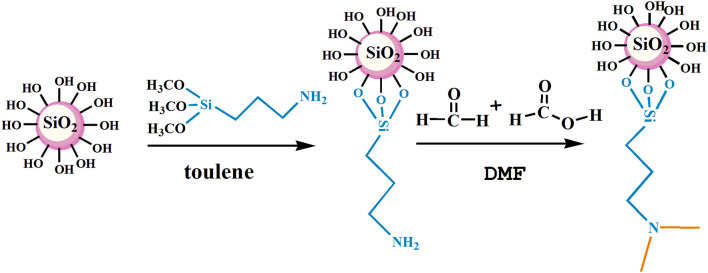
The synthesis route of responsive nano-SiO_2_.

The nano-SiO_2_ was modified by KH540 and then used as the matrix material for synthesizing branched nanomaterials with a tertiary amine group via methylation based on formic acid and formaldehyde. Firstly, 5 g SiO_2_ nano-SiO_2_ was weighed into a 250-mL round flask, followed by the addition of 80 mL methylbenzene as a solvent. KH540 was then dispersed in the methylbenzene solution until homogeneous reaction by constant stirring, and it was then refluxed at 80°C for about 12 h. Subsequently, the mixture was allowed to cool at room temperature for 2 h and then subjected to vacuum filtration and washing with ethanol at least three times until all residues were removed. The powder product was dried at 80°C for 24 h in a drying oven. Secondly, 1 g modified nano-SiO_2_ was scattered in DMF and subjected to ultrasonic treatment in a 250-mL round flask. While stirring, appropriate amounts of formic acid and formaldehyde were introduced and refluxed. Following that, the mixture was cooled to room temperature, and then, using vacuum filtering, washed at least three times with ethanol until all residues were removed. The power product was then dried at 80°C for 24 h in a drying oven. The experimental condition optimization process and the method for determining amine content is present in the Supplementary Materials (the hydroxyl content by thermogravimetric analysis is shown in Figure S1, the optimization of the amount of KH540 by the degree of surface modification of nano SiO_2_ is shown in Table S1, and the relationship between tertiary amine content and reaction conditions is shown in Table S2). The conditions of the responsive nano-SiO_2_ reaction are presented in Table 1. The reaction route of NS–NR_2_ is shown in Figure 1.

**Table 1:** Conditions of responsive nano-SiO_2_.

**Time (h)**	**Temperature (^**°**^C)**	**NS-NH_**2**_: HCOOH: HCHO (g:mol:mol)**	**Solvent (mL)**
12	88	1:8.5:6	70

Responsive nanofluid (0.1 wt%) was obtained as follows. Firstly, 100 mL of distilled water was added to a beaker, and then 0.01 g of PEG-400 was added. The solution was then stirred in a water bath at 50°C for ~10 min. Then, 0.1 g of NaOH was added to the solution, after which 0.1 g of responsive nano-SiO_2_ was slowly added to the solution. When the temperature of the water bath had increased to 80°C, the nanofluid was obtained after about 20 min. The raw nano-SiO_2_ dispersion was also readied in consistency with the above approach.

### Characterization

Infrared (IR) spectra were obtained using the KBr method using a WQF520 spectrometer. ^1^H-nuclear magnetic resonance (^1^H-NMR) spectra were recorded under a Bruker AVANCE III 400 spectrometer (400 MHz) with methanol-D4 solvent. The microtopography of responsive nano-SiO_2_ was characterized using an electron microscope ZEISS Libra 200 FE. The hydrodynamic diameter and proportion of the nanoparticles were determined with a BI 200SM wide-angle dynamic light scattering (DLS) instrument (the details of the DLS measurements are shown in the Supplementary Materials).

### CO_2_/N_2_ Response Tests

A volume of 50 mL of responsive nanofluid (0.1 wt % nanoparticles in water) was transferred to a 125-mL bubbling device with a sand plate as a bubble distributor. This was followed by bubbling with CO_2_ for 20 min and then bubbling with N_2_ for 15 min, during which the behavior of the nanofluid was observed. Flow of CO_2_/N_2_ was controlled at 100 mL/min using a flowmeter. Simultaneously, after bubbling with CO_2_/N_2_, the pH, conductivity, and Zeta potential of the responsive nanofluid were monitored using a pH meter (PB-10), conductivity meter (DDS-307A), and Zeta potentiometer (Zeta PALS 190 Plus), respectively.

### Plugging Experiment

**Figure 2:**
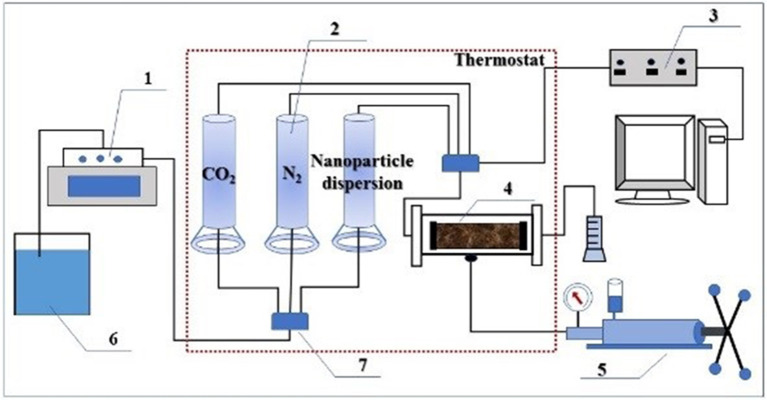
Schematic representation of experimental setup. 1- ISCO pump; 2- middleware; 3- pressure sensor; 4- core holder; 5- hand pump; 6- beaker; 7- six-way valve.

The plugging behavior of the responsive nanofluid was tested in natural core 1# (Table 2) at 45°C. The schematic diagram of the experimental device is shown in Figure 2. CO_2_ flooding was performed at a flow rate of 2 mL/min until the injection pressure stabilized (variation of <1 × 10^−5^ MPa). N_2_ was injected into the core at a flow rate of 1 mL/min, and then responsive nanofluid (saturated absorption CO_2_) was injected into the core at a flow rate of 0.05 mL/min. N_2_ and responsive nanofluid were injected alternately three times, followed by 3-h aging of the core. The second CO_2_ flooding was implemented at a flow rate of 2 mL/min until the injection pressure stabilized (variation of <1 × 10^−5^ MPa). Throughout experiment, the confining pressure was set to 10 MPa, and the difference in the injection pressure was recorded. The plugging efficiency is calculated as,

$$\begin{array}{l} \eta =\frac{K_{1}-K_{2}}{K_{2}}=\frac{P_{1}-P_{2}}{P_{2}} \end{array}$$

where *P*_1_ (MPa) and *P*_2_ (MPa) are the stable pressure of first and second CO_2_ flooding, respectively; *K*_1_ (×10^−3^ μm^2^) and *K*_2_ ( ×10^−3^ μm^2^) are the permeability of the core before and after injecting responsive nanofluid, respectively.

**Table 2:** Basic physical parameters of ultra-low permeability cores.

**Core no**.	**Diameter (cm)**	**Length (cm)**	**Permeability (μm^**2**^ × 10^**−3**^)**	**Porosity (%)**	**Original oil saturation (%)**
1#	3.807	5.013	3.3	11.8	
2#	3.824	5.002	6.4	15.3	54.4
3#	3.815	5.007	7.6	16.5	53.7

### Core Flooding Experiments

The basic physical parameters of core 2# and core 3# are presented in Table 2. The designed core confining pressure was 10 MPa, and the experimental temperature was 45°C. The experiments were conducted as follows. The core was vacuum dried at 100°C for 24 h and was then weighed to find the dry weight. Brine (5,000 mg/L NaCl) was injected into the core at a constant rate of 0.2 ml/min. The displacement pressure was recorded until the pressure generated through the core was stable. The core was weighed to give the wet weight. The prepared crude oil was injected into the brine-saturated core at the constant rate of 0.2 ml/min until there was no brine production, and the irreducible oil saturation was readily obtained by the volume of displaced brine. CO_2_ flooding was performed at a flow rate of 2 mL/min until the pressure became stable. N_2_ was then injected into the core at a flow rate of 1 mL/min, and then responsive nanofluid (saturated absorption CO_2_) or surfactant solution (without responsive nanoparticles) was injected into the core at a flow rate of 0.05 mL/min. To make sure that N_2_ fully interacted with the nanofluid, three cycles of alternating N_2_-nanofluid injection were carried out, followed by 3 h of core aging. The second CO_2_ flooding was performed at a flow rate of 2 mL/min until the pressure was stable. During the experiment, the oil production was recorded.

### Contact Angle and Interfacial Tension Measurements

A clean core slide, however, is strongly water-wet. Hence, in order to simulate an oil reservoir, it was necessary to introduce a lipophilic core surface. Core slides were treated with alcohol and distilled water and then dried in an oven 50°C for 24 h. Following that, core slides were put in the crude oil at 50°C for 10 days to form oil-wet surfaces (initial). According to the wettability alteration to evaluate the efficiency of responsive nanoparticles, the oil-wet core slides were put in responsive nanofluid (0.1 wt%) and surfactant solution for 2 h, respectively. Subsequently, the degree of wettability alteration was determined by measuring a drop of water on the surfaces of treated core slides in air using a KRUSS DSA30S. In addition, the interfacial tension (IFT) between air and aqueous solutions was measured by KRUSS DSA30S. IFT values were obtained using the Young-Laplace equation.

## Results and Discussion

### Characterization

**Figure 3:**
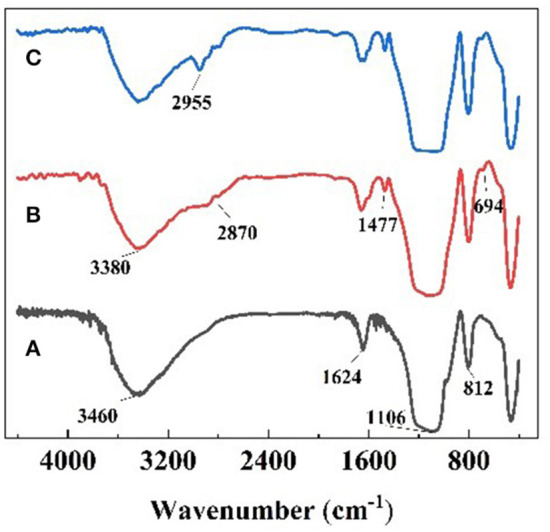
FT-IR spectra of **(A)** raw SiO_2_, **(B)** modified SiO_2_, and **(C)** responsive nano-SiO_2_.

FT-IR spectra of nanoparticles are shown in Figure 3. In the FT-IR spectrum of raw SiO_2_ (a), the strong absorption peaks at around 3,460 and 1,624 cm^−1^ are attributable to the -O-H bonds on the surface of silica. The absorption peaks near 1,106 and 812 cm^−1^ are the asymmetric and symmetric stretching vibration peaks of the Si-O-Si group, which are the characteristic absorption peaks of SiO_2_. In Figure 3B, the new absorption peak at around 2,870 cm^−1^ is the characteristic absorption peak of -CH_2_-, the absorption peak at around 3,380 cm^−1^ is attributable to -N-H stretching, the absorption peak of 1,477 cm^−1^ is attributable to C-N, and the 694 cm^−1^ is absorption peak of Si-C, which elucidated that primary amine was present on the surface of the SiO_2_ due to KH540 modification. In Figure 3C, the new peak area at around 2,955 cm^−1^ is the characteristic absorption peak of -CH_3_, indicating that -(CH_2_)_3_NH_2_ had reacted to be -(CH_2_)_3_N(CH_3_)_2_ via the Eschweiler-Clarke method.

**Figure 4:**
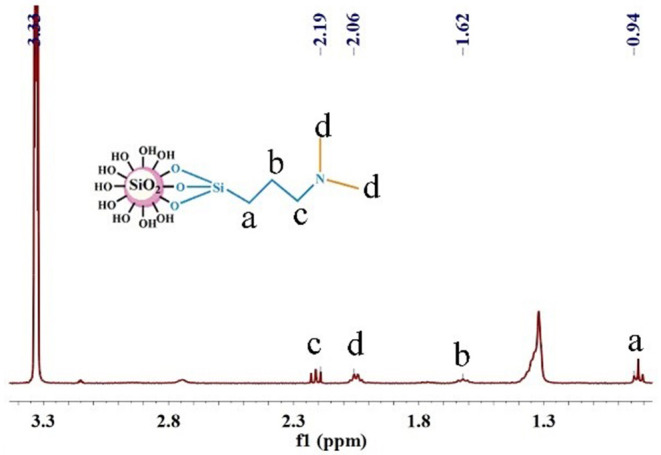
^1^H NMR spectra of responsive nano-SiO_2_, using CD_3_OD as a solvent.

As shown in Figure 4, the signal of the protons in -Si-C**H**_2_- was 0.94 ppm, the signal of the protons in -C**H**_2_- was 1.62 ppm, the signal of the protons in -C**H**_3_ was 2.06 ppm, and the signal of the protons in -C**H**_2_-N- was 2.19 ppm, which implied that the structure of the surface on nano-SiO_2_ was consistent with that expected of the responsive nano-SiO_2_ structure.

**Figure 5:**
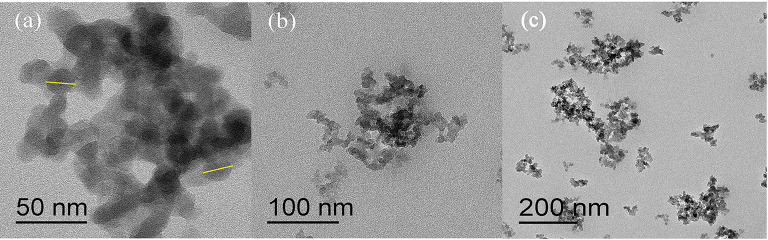
TEM image of responsive nano-SiO_2_. The scale bars in **(a–c)** are 50, 100, and 200 nm, respectively.

The microscopic structure formed by responsive nano-SiO_2_ was observed from TEM morphology, as shown in Figure 5. Responsive nano-SiO_2_ with a size of 20 nm aggregated slightly due to the particle size being in the nanometer scale and its high surface area.

### CO_2_/N_2_ Response

**Figure 6:**
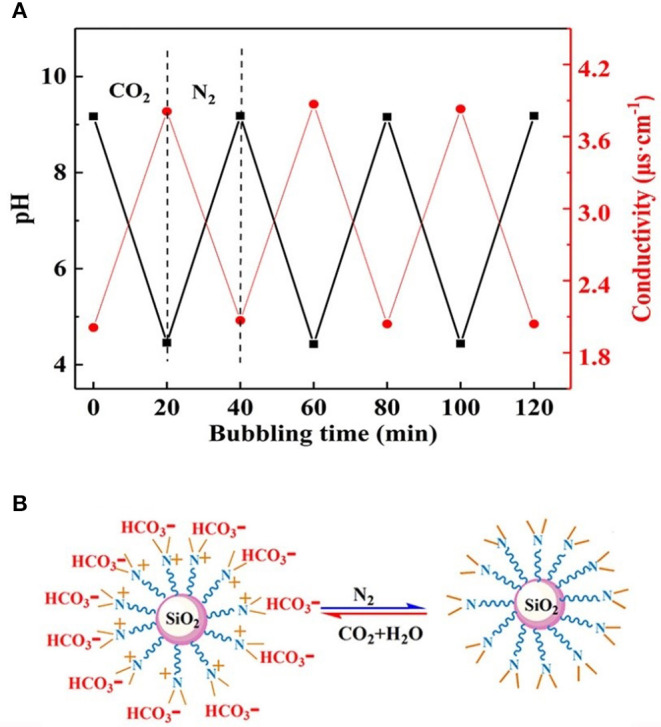
**(A)** Changes in pH and conductivity under alternate bubbling of CO_2_ and N_2_. **(B)** Protonation and deprotonation of nanoparticles by bubbling CO_2_ and N_2_.

**Figure 7:**
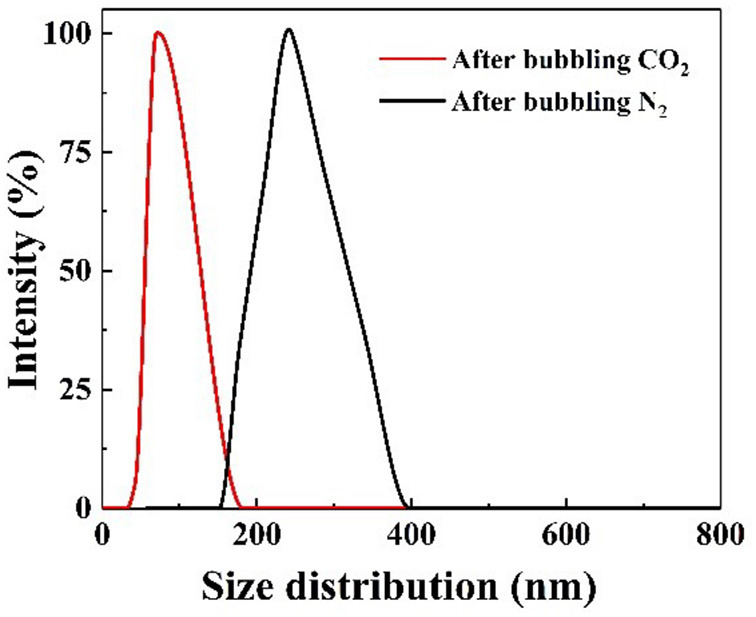
Diameter distribution of nanoparticles in dispersion before and after bubbling CO_2_/N_2_.

To confirm whether the CO_2_/N_2_ trigger is a reversible transformation, the effects of CO_2_/N_2_ on the pH and conductivity of the prepared responsive nanofluid were investigated. As shown in Figure 6A, CO_2_ and N_2_ were bubbled into nanofluid, respectively. When CO_2_ bubbled into the nanofluid, its pH decreased to 4.4 and its conductivity increased to 3.9 μs·cm^−1^. While N_2_ was bubbled into nanofluid, its pH rose back to 9.1 and its conductivity decreased back to 2.1 μs·cm^−1^. It could be seen that responsive nanofluid experienced a cyclical variation in pH and conductivity. This variation was attributed to the protonation and deprotonation of the tertiary amine group on the surface of nano-SiO_2_. The responsive process is illustrated in Figure 6B. The responsivity tests showed that the tertiary amine groups could interact with CO_2_/N_2_, which could be used to control the properties of nanofluid such as in CO_2_-triggered switchable surfactants reported by Liang et al. (2011), CO_2_-sensitive foams researched by Li et al. (2016), or CO_2_-triggered gelation proposed by Li et al. (2017). However, this paper intends to control the stability of nanoparticles in nanofluid via CO_2_/N_2_ response. Interestingly, upon bubbling CO_2_ through the responsive nanofluid, it remained a clear and transparent dispersion, while when N_2_ was bubbled into this nanofluid for 5 min, a dramatic change occurred immediately: the nanofluid changed from a clear and transparent dispersion to a milky suspension, as shown in Figure S3. When responsive nanoparticles being replaced with raw SiO_2_, the nanofluid always remained clear and transparent while bubbling in CO_2_/N_2_. This result indicated that responsive nanoparticles with tertiary amine played an important role in the CO_2_/N_2_-responsive behavior. Such nanoparticles could be stably dispersed in surfactant solution via steric hindrance or the electrostatic repulsive force among them. After bubbling CO_2_ into dispersion, the Zeta potential was measured to be 22.45 mV, indicating that positive charges occurred on the surface of nanoparticles due to protonation of the tertiary amine, and the electrostatic interaction between nanoparticles could still hinder nanoparticle agglomeration. After bubbling in N_2_, the −8.47 mV Zeta potential suggested negative charges on the surface of nanoparticles due to deprotonation of the tertiary amine and that electrostatic interaction between nanoparticles was weakened, resulting in nanoparticle aggregation. In summary, it can be inferred that the deprotonated tertiary amine groups resulted in a decrease in electrostatic repulsive force among nanoparticles. The DLS analysis (Figure 7) indicated that the hydrodynamic diameter of the nanoparticles, which had a uniform size distribution, was ~60 and 245 nm after bubbling in CO_2_ and N_2_, respectively. Therefore, it may be feasible to block the gas-channeling channels with particle aggregates generated by the reaction of responsive nanofluid with CO_2_/N_2_. Moreover, N_2_ and CO_2_ do not lead to pollution of the formation and are also used as displacement agents, so they can be regarded as eco-friendly triggers.

### Plugging Experiment

**Figure 8:**
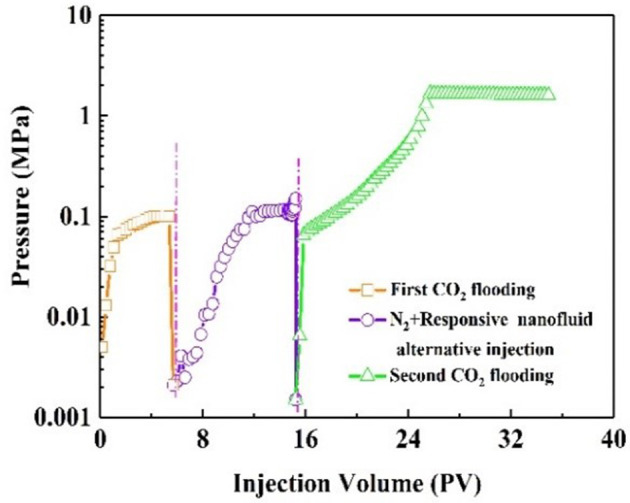
Change of pressure during the core #1 plugging experiment.

The dominant channel provides the flow routes of CO_2_ gas breakthrough (Zhang et al., 2019). As shown in Figure 8, the injection pressure increased quickly and finally stabilized at 0.1 Mpa during the first CO_2_ flooding. At this point, gas channeling had occurred. A certain amount of N_2_ was injected into the core and played a role in the CO_2_ response in the subsequent operational process. Following that, the saturated CO_2_ responsive nanofluid was injected into the core. After N_2_ and nanofluid injection, the pressure was not obviously increased. After aging for 3 h, the CO_2_ was injected again. During the second CO_2_ flooding, the injection pressure increased gradually then stabilized at 1.5 MPa, indicating that responsive nanoparticles could effectively plug the gas channel in the core. The plugging efficiency for the core was 93.3%. The responsive nanoparticles thus showed plugging capacity in ultra-low permeability reservoirs.

### Core Flooding Experiments

**Figure 9:**
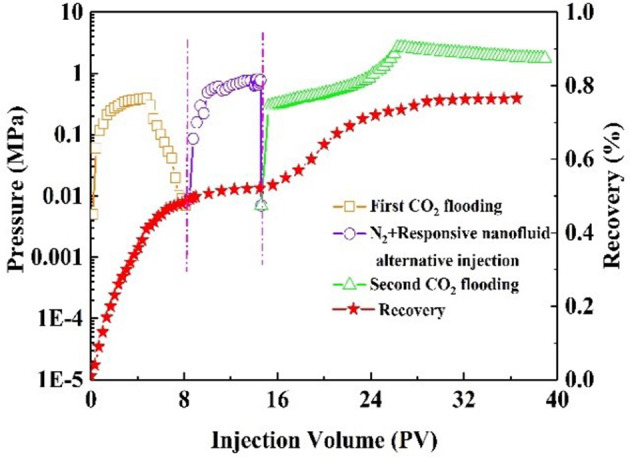
Pressure and recovery factor during the core #2 flooding experiment (purple line: N_2_ and responsive nanofluid injection).

**Figure 10:**
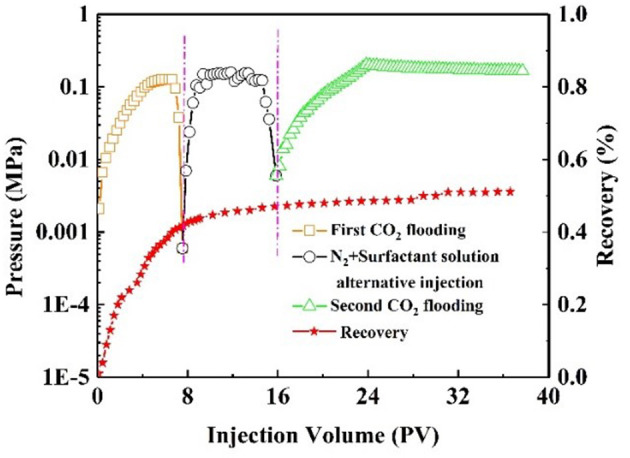
Pressure and recovery factor during the core #3 flooding experiment (black line: alternate N_2_ and surfactant solution injection).

Oil recovery and pressure recording are shown in Figure 9. At the initial stage of CO_2_ injection into the core, the pressure increased rapidly. The resistance to CO_2_ inflow into the core was gradually increasing. When the pressure had increased to 0.14 MPa, it began to decline, indicating CO_2_ breakthrough. As a result, CO_2_ bypassed the oil zone, leaving a large amount of oil in the core. The recovery factor was 50% of the original oil in place at this stage. After alternating injection of N_2_ and nanofluid, the injection pressure increased and finally stabilized. The core was aged for 3 h. During the second CO_2_ flooding, the injection pressure increased, subsequently reaching maximum, and then stabilized at 2.1 MPa. The recovery factor increased by 26%. Comparatively, with slug injection with the same experimental procedure, the change in injection pressure was slight (Figure 10). The total oil recovery was 51%, and only 3% of original oil in place bypassed by the first CO_2_ flooding was recovered. It can be seen that responsive nanoparticles play a vital role in enhanced oil recovery.

### EOR Mechanisms

**Figure 11:**
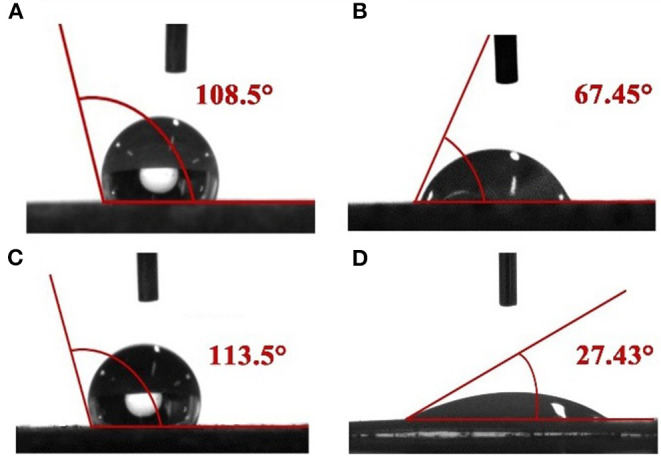
Contact angles of water on a core slide before **(A,C)** and after treatment with surfactant solution (without nanoparticles) **(B)** and responsive nanofluid **(D)**.

**Figure 12:**
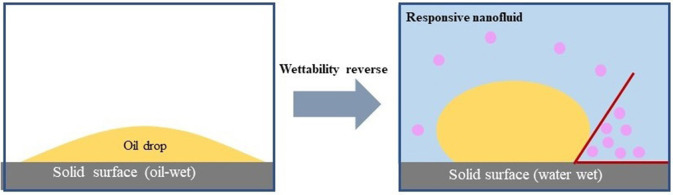
Mechanism of wettability alteration induced by the responsive nanofluid (Wasan et al., 2011).

The effect of responsive nano-SiO_2_ on wettability was tested. After being soaked in crude oil, the surface of the rock exhibited an oil-wet state (Figures 11A,C). Figures 11B,D show the contact angles measured after exposure to surfactant solution (without nanoparticles) and responsive nanofluid, respectively. It was observed that responsive nanoparticles could effectively change the wettability of the core surface. This might be associated with the adsorptive behavior of the nanoparticles on the core surface. Wasan and Nikolov (2003) and Kondiparty et al. (2011) reported that the presence of nanoparticles in the three-phase contact region increases the tendency to create a liquid wedge-film. Moreover, this wedge-film separates formation fluid such as oil, paraffin, water, and gas from the formation surface. In this case, based on Figure 11, a mechanism is proposed in Figure 12 to interpret the wettability alteration induced by responsive nanoparticles. As a result of the electrostatic and Vander Waals forces, responsive nanoparticles “wedge spread” on the core surfaces, and thus the oil drop adsorbed on the surface was detached.

**Figure 13:**
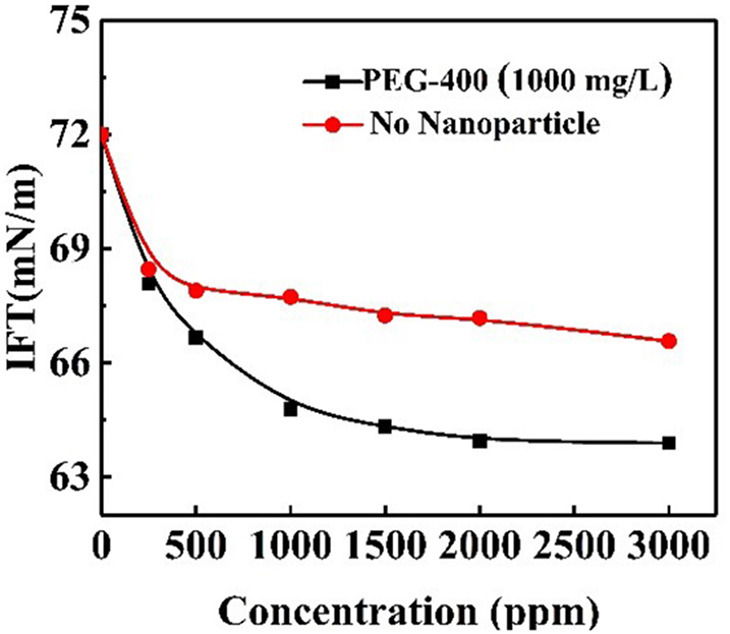
Air–water interfacial tensions for responsive nanoparticle-augmented surfactant solution of different surfactant concentrations at constant nanoparticle concentration.

The IFT values for PEG-400 surfactant solutions of different concentrations with the addition of responsive nanoparticles were obtained. The nanoparticle concentration was equivalent to 1,000 mg/L for all solutions. The interfacial effects of responsive nanoparticles when mixed with surfactant solution are shown in Figure 13. In the presence of nanoparticles, IFT decreased more steeply at concentrations below 1,000 mg/L. Moreover, IFT was lower for almost all surfactant concentrations when compared to the system without nanoparticles. The co-adsorption of responsive nanoparticles and surfactants at the interface could lead to the lower IFT. However, it should be pointed out that responsive nanoparticles had no obvious effect of reducing IFT.

During the CO_2_ flooding process, CO_2_ can greatly reduce oil viscosity to enhance oil flow. Additionally, CO_2_ can dissolve colloids in the reservoir to increase permeability. The molecular diffusion of CO_2_ promotes the penetration of CO_2_ into the reservoir (Yu et al., 2015; Lashgari et al., 2019). As a consequence, oil is recovered via CO_2_ flooding. However, CO_2_ gas channeling in reservoirs can result in low sweep efficiency, and a large quantity of oil may remain in the reservoir (Gong and Gu, 2015; Naderi and Simjoo, 2019). To address this, responsive nanofluid and N_2_ were subsequently alternately injected and reacted in the breakthrough channel. After aging for 3 h, nanoparticles slowly agglomerated in contact with N_2_, which can effectively block the breakthrough channel to control CO_2_ mobility. CO_2_ was almost forced into the lower permeability zone with remaining oil during the second CO_2_ flooding. The remaining oil was widely swept from the reservoir. Thus, displacement efficiency was increased through CO_2_ mobility control, and perhaps the wettability alteration of responsive nanoparticles without contact with N_2_ also contributed to enhance oil recovery. In summary, responsive nanoparticles may be used as an ideal CO_2_ channeling blocking agent and displacement agent in ultra-low permeability reservoirs.

## Conclusion

In this work, surface-modified SiO_2_ nanoparticle-based nanofluid was investigated for EOR. The performances of responsive nanoparticles, including CO_2_/N_2_ response, wettability alteration, interfacial behavior, displacement behavior, etc., were examined. Responsive nanoparticles exhibited a good CO_2_/N_2_ response by bubbling in CO_2_/N_2_ to control nanoparticle dispersity due to electrostatic interaction. An outstanding plugging capacity of 93% was achieved in the plugging experiment. Core flooding experiments indicated that 26% of oil recovery was achieved by responsive nanoparticles in an ultra-low permeability reservoir. Controlling CO_2_ mobility was the primary mechanism of EOR. In addition, through surface adsorption, responsive nanoparticles reverted oil-wet surfaces toward the water-wet state, which was helpful for improving oil displacement efficiency. The results indicated that responsive nanoparticles may have a high potential to enhance oil recovery during CO_2_ flooding in ultra-low permeability reservoirs.

## Data Availability Statement

All datasets generated for this study are included in the article/Supplementary Material.

## Author Contributions

NL conceived the idea and supervised the research work overall. QZ and GC contributed to the experiment methods and data analysis. QZ wrote the manuscript and drew all the figures. DQ and KC came up with ideas for the manuscript. LT and DW contributed to revision of the paper.

## Conflict of Interest

KC was employed by China National Offshore Oil Corporation (CNOOC) Energy Development Company Limited, Tianjin, China. The remaining authors declare that the research was conducted in the absence of any commercial or financial relationships that could be construed as a potential conflict of interest.
