# Supplementary material for: CO2/N2-Responsive Nanoparticles for Enhanced Oil Recovery During CO2 Flooding
Source: Front Chem. 2020 May 21;8:393. doi: 10.3389/fchem.2020.00393 (PMC7253667; doi:10.3389/fchem.2020.00393)
Supplement: Supplementary file 1 [file Data_Sheet_1.docx]

Supplementary Material

**The hydroxyl content on surface of nano-SiO_2_ was measured by** **thermogravimetric analysis** (Netzsch Scientific Instruments).

Three hydroxyl groups that in different states on the surface of nano-SiO_2_ can be removed at different temperatures. Therefore, the weight loss of nano SiO_2_ was measured at different temperatures, the content of surface silicon hydroxyl groups could be determined. As shown in Figure S1, 7.22% of weight was loss on the surface of nano-SiO_2_ after heating up to 900 °C, the hydroxyl content on the surface of nano-SiO_2_ can be calculated as 4.24 mmol/g.


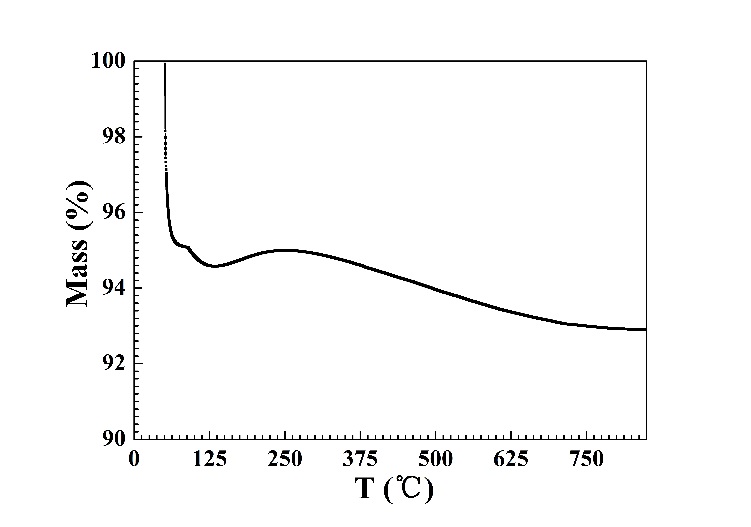


**Supplementary Figure 1.** The thermogravimetric curve of nano-SiO_2_.

**The method of determining the content of amine groups**

The yield rate of modified nanoparticles is calculated by the potentiometric titration method. Crystal violet was used as an indicator and perchloric acid–glacial acetic acid was used as the standard liquid. Crystal violet was used as an indicator and perchloric acid-glacial acetic acid was used as the standard solution. The yield rate of responsive nanoparticles is calculated by same method, but it is worth noting that adding anhydride promoted unreacted primary amine groups undergo acetylation in sample dispersion. For example, the potentiometric titration curve of sample dispersion as shown in Figure S2. In Figure S2a the color of the dispersion changed from from purple to blue to green with the increase of standard solution, The point where the diapersion turns blue is the end point. However, the point where the diapersion turns blue is difficult to determine. Second derivative of the curve was obtained in Figure S2b, the point corresponding to the zero point is the end point.


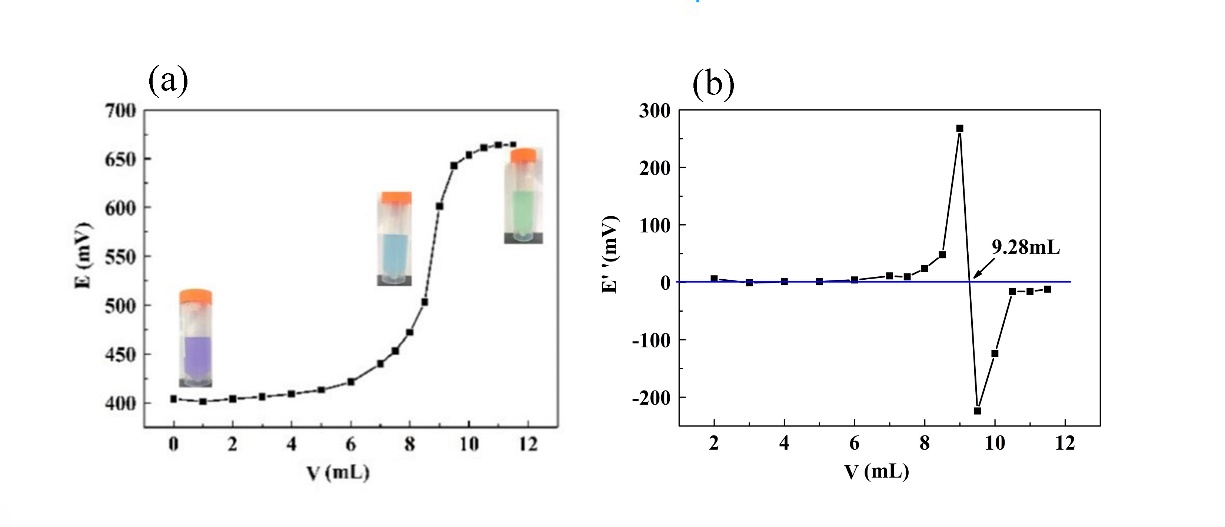


**Supplementary Figure 2.** (a) The potentiometric titration curve; (b) Second order differential of potentiometric titration curve.

The amino groups content was calculated by the following formula

| $X=\frac{F(V_{1}-V_{2})}{m}$ |  |
| --- | --- |

Where, *X* refers to the content of amino group in the sample (mmol/g), *m* is the mass of the sample (g), *F* is the standard concentration of the perchloric acid–glacial acetic acid solution (mol/mL), *V_1_* is the volum of standard solution for the sample (mL), *V_2_* is the the volum of standard solution for the blank control group (mL).

**Experimental condition optimization**

The surface modification degree of nano-SiO_2_ under different KH540 dosage is shown in the Table S1. When the dosage of KH540 was 2500μL, the modification degree of nanoparticles reached 98.85%. The optimized conditions of responsive nano-SiO_2_ synthesis are shown in Table S2.

**Supplementary Table 1.** Effect of KH-540 dosage on surface modification degree of nano-SiO_2_.

| Nano-SiO_2_（g） | KH-540（μL） | Primary amine groups（mmol/g） | Modification degree（%） |
| --- | --- | --- | --- |
| 5 | 760 | 0.6953 | 49.51% |
| 5 | 1400 | 0.9538 | 67.92% |
| 5 | 1600 | 1.0229 | 72.84% |
| 5 | 2500 | 1.3881 | 98.85% |
| 5 | 3000 | 0.9733 | 69.30% |

**Supplementary Table 2**. Relationship between tertiary amine content and reaction conditions.

| Solvent (mL) | NS-NH_2_: HCOOH: HCHO (g:mol:mol) | Temperature (ºC) | Time (h) | Tertiary amine content (mmol/g) |
| --- | --- | --- | --- | --- |
| 60 | 1:7:6 | 90 | 12 | 0.9367 |
| 65 | 1:7:6 | 90 | 12 | 0.9603 |
| 70 | 1:7:6 | 90 | 12 | 1.065 |
| 75 | 1:7:6 | 90 | 12 | 1.002 |
| 80 | 1:7:6 | 90 | 12 | 0.9397 |
| 70 | 1:7.5:6 | 90 | 12 | 1.092 |
| 70 | 1:8:6 | 90 | 12 | 1.116 |
| 70 | 1:8.5:6 | 90 | 12 | 1.141 |
| 70 | 1:9:6 | 90 | 12 | 1.03 |
| 70 | 1:8.5:6 | 84 | 12 | 1.015 |
| 70 | 1:8.5:6 | 86 | 12 | 1.068 |
| 70 | 1:8.5:6 | 88 | 12 | 1.153 |
| 70 | 1:8.5:6 | 92 | 12 | 1.076 |
| 70 | 1:8.5:6 | 88 | 10 | 1.026 |
| 70 | 1:8.5:6 | 88 | 11 | 1.075 |
| 70 | 1:8.5:6 | 88 | 13 | 1.123 |
| 70 | 1:8.5:6 | 88 | 14 | 1.013 |

Details of the DLS measurements

After the CO_2_ is pumped into the nanofluid, a portion of the nanofluid is moved to a specific vial for testing with a BI-200SM wide-angle dynamic laser light scattering. Then, the N_2_ is pumped into the nanofluid, and a portion of the liquid is moved to a specific vial for testing with a dynamic laser light scattering instrument. Data including the mean diameter, intensity distribution, and cumulative distribution are obtained. According to the test data, a graph is drawn to get Figure 7.


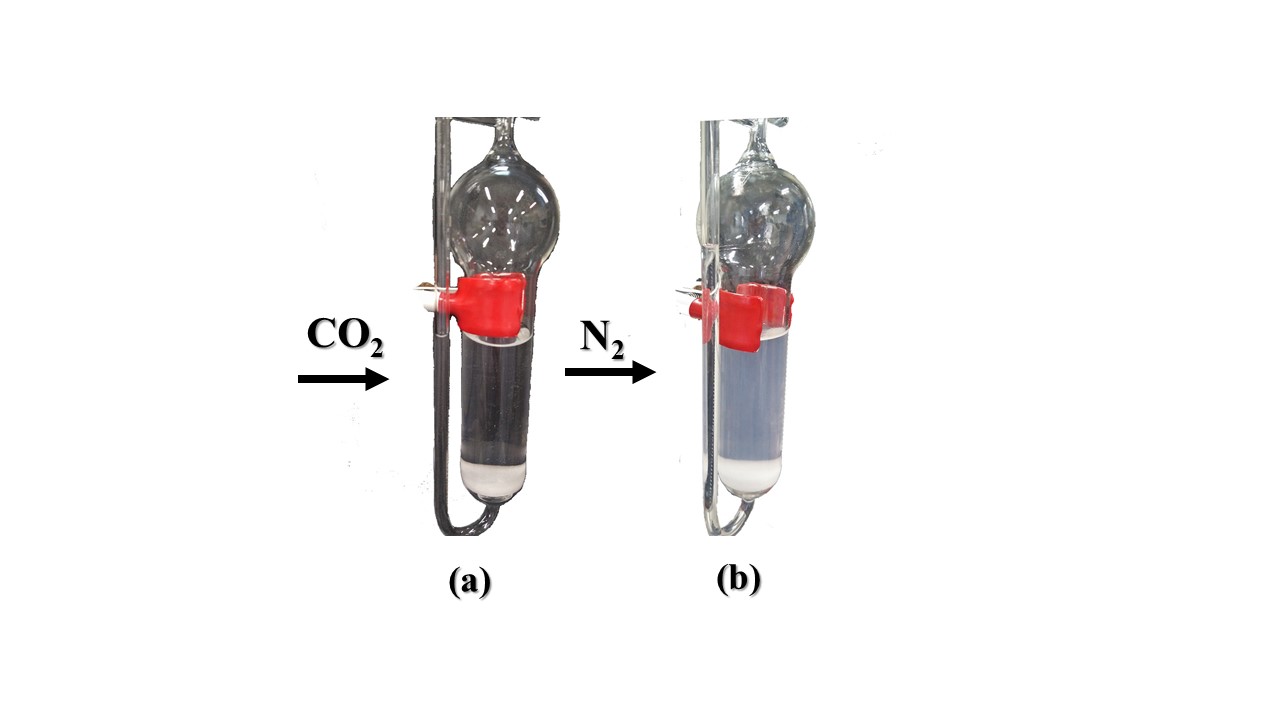


**Supplementary Figure 3.** (a) after bubbling CO_2_ through the nanofluid within 15 min, it exhibits clear and transparent solution and (b) after bubbling N_2_ for 5 min, it exhibits milky suspensions.
